# Moroccan suicidal schizophrenics: Case study in arrazi hospital of sale

**DOI:** 10.1192/j.eurpsy.2023.2292

**Published:** 2023-07-19

**Authors:** N. El Moussaoui, S. Bahetta

**Affiliations:** Psychiatry, Arrazi Hospital of Sale, Salé, Morocco

## Abstract

**Introduction:**

Schizophrenia is a severe, common, chronic mental disorder with a prolonged and disabling course, having a high social impact.

Mortality is two to three times higher in schizophrenic patients than in the general population.

Suicide is the main cause of death in patients with schizophrenia. In spite of great efforts in preventing such deaths, suicide rates have remained alarmingly high, highlighting the need for a better understanding of the phenomenon.

**Objectives:**

The objective of this work is to determine the prevalence of suicide in schizophrenic patients, to investigate the main risk factors in these patients and the characteristics of suicide and the therapeutic management of the patients.

**Methods:**

This is a retrospective study on medical records about 43 patients (32 men / 11 women) who were admitted to the Arrazi Hospital in Salé, from september 2021 to september 2022, using an operating form grouping socio-demographic criteria of the patients, personal and family history, characteristics of the suicide attempt and management.

**Results:**

In this study, 75% were male and 25% were female with an average age of 34.5 years. The existence of a personal history of suicidal ideation, plans and attempts is a major risk factor for suicide. The lethality of the means used reflects a higher degree of suicidal intentionality. Clozapine, in particular, plays a protective role by reducing the rate of suicides and suicide attempts.

**Conclusions:**

Despite therapeutic progress, the prevalence of suicide among patients suffering from schizophrenia is still high.

The prevention of suicide in these patients remains fundamental, as does the reduction of positive or negative symptoms, the improvement of quality of life, the reduction of the handicap caused by this illness and the fight against the stigmatization of patients.

**Disclosure of Interest:**

None Declared

